# Reports on the Hospitals of the United Kingdom

**Published:** 1910-04-09

**Authors:** Henry Burdett


### REPORTS
ON THE
### Hospitals of the United Kingdom.

By SIR HENRY BURDETT, K.C.B., K.C.V.O.

### XXXVIII.—THE MANCHESTER ROYAL INFIRMARY.

The history of this institution is as remarkable as it is interesting. It is probably thirty years ago since we first had a hand in its affairs, when the possibility was considered of reconstructing the old hospital without rebuilding. Many things have happened since then, and memory recalls a number of members of the profession and Manchester merchants to whom the old infirmary was very precious. When it was determined to rebuild on the Stanley Grove site, it was decided to arrange that a large sum of money should be available to defray the cost of a great hospital containing 600 beds. This new hospital presents a noble appearance when viewed from the roadway, for the elevation is strikingly attractive, and cannot fail to excite the just pride of the citizens of Manchester.

### Modern Hospital Planning.

It is not easy for the most expert and experienced of men to sit down and plan a hospital of this size, which shall provide everything material in accordance with the most modern developments, and at the same time prove when built to be up-to-date in every respect, if it is not even in some sense the embodiment of new principles which will mark a new departure and become crystallised as improved units of construction for the admiration and adoption of all who come after. When the planning of such a hospital is entrusted to an architect, however able, and especially when his main work has been in other directions than those of a clinical hospital, it would be on all accounts best for him and juster to the great interests which are entrusted to his hands, to associate with him some men, or one man at least, whose intimate knowledge of the requirements of a modern clinical hospital would render it certain that each section of the plans ultimately submitted should be complete and satisfactory. Nearly all the errors, the large cost, and the growing tendency to increase the cost of modern hospital buildings are due to the non-adoption of the course which is here suggested. How is it possible to expect that any architect, without such technical co-operation, can evolve on his own initiative a perfect modern hospital of the Manchester Infirmary type? Much remains to be done in the matter of the preparation of plans and in the selection of the people to whose hands they may best be entrusted at the outset, so as to avoid the frequent failure to secure adequate safeguards in connection with the appointment of assessors in the case of limited competitions. Everybody concerned with the administration of hospitals should in future make it his business to become familiar with the causes of the failures and disappointments in recently erected great hospitals which may have resulted from an absence of knowledge and control in regard to the preparation and selection of plans. In this way only can we hope to see the time when an effective check will be put upon the excessive cost per bed, the frequent omissions, and the reproduction of many faults in existing plans that would never have occurred had proper wisdom been displayed by the representatives of the people who subscribed the money for this class of building. We are quite certain that despite many things which are good, and much which is commendable in regard to the arrangements of the new Royal Infirmary at Manchester, no one will fail to grasp that the plans would certainly have been better than they, in fact, are, had the course we have indicated been followed at the outset. It is fair to add that, at the time the plans were settled, the association of an outside lay expert with the assessor was not the general practice, as it is likely to be in future.

### The New Buildings.

We do not propose to attempt any description of the buildings as such, for the sufficient reason that we published a full account of them with plans on page 99 of The Hospital for October 24, 1908. One special feature of the plan is the provision of covered ways, one of which—namely, that extending to the south boundary of the site—is no less than 756 feet long. From this main way branches extend, with staircases and electric lifts; that to the south gives access to all the medical pavilions, and that to the north to all the surgical pavilions. All the internal traffic of the hospital by means of these ways is under cover. The sides of these ways, however, are only enclosed to a height of four feet, so that the air has free access everywhere, whilst the ways are protected by the sides and overhanging roof from the inclemency of the weather. We had an opportunity of testing the effectiveness of this overhanging roof, which projects about 2¼ feet, and we found it stood the test well. It no doubt involves certain risks of cold and illness for those members of the staff who have most frequently to be in these covered ways in all weathers, but if the practice of providing free air spaces between each part of the hospital is an essential condition of modern hospital construction, the corridors as they stand may be thought satisfactory. In certain ways they are not satisfactory, and never can be in the hygienic and medical sense, for the theatres are so constructed that patients, after undergoing an operation, however prolonged, must be brought out into these cold and open corridors in all weathers on their return from the theatre to the wards. It is true that the distance to be traversed may be small, which diminishes the risk, but risk there must be, and it is a risk which ought to be provided against in the original planning of a great hospital like this, so as to fulfil the requirements of modern surgery and to provide the maximum of security to every patient operated upon.

### The Wards.

The good points about the plan are the wards, the grouping of the administration buildings, the pathological department, the situation selected for the out-patient department, the laundry and the workshops, and the septic pavilion. The wards are well proportioned, well lighted, and well planned in regard to cubic and superficial area. Each rectangular unit contains 24 beds as a maximum and 20 as a minimum; the circular wards provide accommodation for 16 beds each. We are glad that some circular wards were included, as they lend themselves naturally to the site, and are attractive and good. One new feature is that each ward unit has a day-room or class-room, as it is called, which is situated apart from the wards off the covered way, so that the patients who are convalescent can spend the day, or some of it, in these rooms outside the wards. This feature, which is novel, was greatly criticised on administrative grounds at the outset, but we were informed that in the working it has proved a useful addition, and that there has been no difficulty as to discipline in practice. It is, of course, very desirable, where a day-room is provided, that it shall be so placed as to be under the direct and easy supervision and control of the sister-in-charge. The plan on which the hospital is built did not admit compliance with this requirement. We consider that the sanitary blocks are too small, an oversight which is too frequently apparent in hospital buildings even of a recent date. Architects have not at present grasped the importance of giving ample space in these blocks to enable the staff to do the work readily with the maximum of expedition and efficiency. To secure this result there must be plenty of space, for its absence makes it difficult or impossible for the work to be undertaken readily by more than one member of the staff at a time. This hospital is one more proof that for fittings the maximum quality is essential to the successful endurance of the strain of excessive wear and tear which such fittings have to undergo in a great hospital where the pressure upon the beds is constant and severe.

One of the most remarkable and novel features of the administration block is the fine board-room provided for the meetings of the medical staff. A further feature of this new Infirmary is the basement, which is very extensive, and provides accommodation for storage to an extent so large as to be almost, if not quite, unique. There is ample storage room, which is essential to so large a hospital. We noted too with pleasure that the general order and arrangement of the stores reflect credit upon all who have to do with their management.

The pathological department, as already stated, is good on the whole, and though some of the fittings in the mortuary section gave trouble at first, they are now completely satisfactory. There is ample space for good and abundant work to be done in this section, and the arrangements provide for the proper fulfilment of every detail with reverence and a due regard for the feelings of the friends of the patients. The isolation block is small, which is a good feature in one sense, though it may ultimately prove inadequate for a great city hospital containing upwards of 550 beds. The out-patient and casualty departments do not contain any features which call for special remark. The provision of a bedroom and sitting-room for the medical officer on duty is to be commended.

### Some Criticisms in Conclusion.

A great hospital of this kind is not easy to administer under the most favourable conditions. Before the buildings were completed we pointed out that in our view it was an error so to plan the buildings that the wards had to be approached by a double corridor. Apart from the addition to the original cost entailed, and the considerable extra annual expenditure for cleaning, etc., a double corridor is administratively objectionable, and ought to be avoided wherever possible in the planning of a modern, up-to-date hospital. It leads to much trouble in the ordinary working of a hospital where it is essential sometimes to be able to find readily an officer on duty in the hospital, and this difficulty has made itself felt and will continue to be felt at Manchester without doubt. Just as it is essential to have one entrance, and one only, for goods and for patients respectively, so it is essential to facilitate the control and supervision of a great institution like this, that the double corridors shall be avoided.

We are glad to hear that the patients are well satisfied with the accommodation provided and the comfort they have experienced in the new Infirmary. The committee showed great wisdom in selecting for the post of superintendent Mr. W. G. Carnt, for the strain and work which devolved upon the general superintendent, the matron, and the lady housekeeper when these new buildings had to be equipped and reduced to order, and due preparation made for the reception of patients, must have been onerous indeed. Work of this kind involves great administrative gifts, great self-denial and mastery of detail, and an imagination capable of realising exactly how to arrange for the work to be commenced at the right end and systematically carried out, so that the utmost possible economy and expedition may result. All this Mr. Carnt, Miss Sparshott, and Mrs. Morris accomplished quietly and well, so that the patients were transferred, without the slightest hitch and in a few hours, from the old infirmary to the new, where they found everything provided for their comfort and well being. We have no doubt that the committee, and the residents of Manchester and the district, fully recognise the excellent service thus rendered, and that they appreciate the smooth and efficient working of this vast series of buildings which has been ably brought about by the devotion and ability of these officers.

It will be a serious task to increase the revenue by some £10,000 a year, a sum which we understand will be required as additional income to enable the new Infirmary to be fully occupied and the work kept at the highest point of efficiency.

As we have said the appearance presented by the hospital as it is approached is striking and attractive. This fact, coupled with the circumstance that we understand that the work of the numerous contractors engaged has turned out to be good and remarkably free from faults, reflects great credit upon the architects, Mr. E. T. Hall and Mr. John Brooke, whose responsibility has been great indeed. That responsibility is increased by the circumstance that the plan really provides on a small site a relatively large village which contains accommodation for the continuous residence of nearly one thousand patients and staff.

The **Worcester General Infirmary** will be reported on next week.

